# Biocompatibility Issues with Modern Implants in Bone - A Review for Clinical Orthopedics

**DOI:** 10.2174/1874325000802010066

**Published:** 2008-04-25

**Authors:** Katja M.R Nuss, Brigitte von Rechenberg

**Affiliations:** Musculoskeletal Research Unit, Vetsuisse Faculty, University of Zurich, Switzerland; Vetsuisse Faculty, Equine Hospital, Winterthurerstrasse 250, Ch-8050 Zurich, Switzerland

## Abstract

Skeletal defects may result from traumatic, infectious, congenital or neoplastic processes and are considered to be a challenge for reconstructive surgery. Although the autologous bone graft is still the “gold standard”, there is continuing demand for bone substitutes because of associated disadvantages, such as limited supply and potential donor side morbidity [1]. This is not only true for indications in orthopedic and craniomaxillofacial surgeries, but also in repairing endodontic defects and in dental implantology.

Before clinical use all new bone substitute materials have to be validated for their osseoconductive and - depending on the composition of the material also –inductive ability, as well as for their long-term biocompatibility in bone. Serving this purpose various *bone healing models* to test osteocompatibility and inflammatory potential of a novel material on one hand and, on the other hand, *non-healing osseous defects* to assess the healing potential of a bone substitute material have been developed. Sometimes the use of more than one implantation site can be helpful to provide a wide range of information about a new material [2].

Important markers for biocompatibility and inflammatory responses are the cell types appearing after the implantation of foreign material. There, especially the role of foreign body giant cells (FBGC) is discussed controversial in the pertinent literature, such that it is not clear whether their presence marks an incompatibility of the biomaterial, or whether it belongs to a normal degradation behavior of modern, resorbable biomaterials.

This publication is highlighting the different views currently existing about the function of FBGC that appear in response to biomaterials at the implantation sites. A short overview of the general classes of biomaterials, where FBGC may appear as cellular response, is added for clarity, but may not be complete.

## BONE SUBSTITUTES

### Autografts

Autografts are still the method of choice to substitute damaged or lost bone. The transplanted bone is largely necrotic and acts as a scaffold for the ingrowth of granulation tissue containing osteoprogenitor cells. Under the influence of local factors (cytokines, etc) these osteoprogenitor cells differentiate into osteoblasts that are responsible for new bone formation. At the same time the graft matrix is resorbed through osteoclasts. This process of graft resorption occurring parallel to bone formation was already discovered by Phemister in 1914 [3], who named it “creeping substitution”.

### Biomaterials as Bone Substitutes

Bone substitute materials increasingly replace the use of autografts and can be divided into three major classes: polymers, ceramics and natural materials. Nowadays they are used either alone or in combinations called composites [4]. Regardless of their composition, once they are implanted in bone, they also undergo the process of creeping substitution, although the speed and rate of this process may vary according to their composition [5].

### Polymers

Synthetic polymers represent the largest class of biomaterials useful in applications in both, soft and hard tissue. They can be hydrophobic like silicone rubber (SR), polypropylene (PP), polyethylene (PE) and polymethylmetacrylate (PMMA), or water swelling or even water soluble like polyethylene glycol (PEG). Some of them are degradable, others remain almost unchanged within the body. Polymers are long chained molecules consisting of a large number of small repeating units [6]. They can be amorphous or semicrystalline and their surfaces may be modified chemically and biochemically.

*PMMA*, to cite the most important polymer in current orthopedics, is a permanent bone substitute material which is frequently used to improve the anchorage of fracture fixation devices and joint replacement prostheses. It is also used in vertebroplasty in severe cases of impact fractures of the vertebral body due to osteoporosis or neoplasm [7, 8]. Although this material has proven its usefulness in these applications, on the other hand it poorly osseointegrates even possibly disturbing bone healing and remodeling through its genuine inert properties. Additionally tissue necrosis may be caused through heat production up to 80°C while curing and by creating monomer toxicity [9, 10]. In combination with primary (micro-)mechanical instability these properties may lead to the formation of an interface membrane and subsequent aseptic loosening [11-13]. Despite all those concerns, PMMA is still the most frequently used polymer bone cement in Europe [14].

Hydrogels are novel polymers that gained more popularity in recent years. As an example for degradable water containing substances they can be injectible and of different water contents. They can consist of Poly-(ethylene glycol) [15], or gelatine [16-18].

They are used experimentally and clinically as biomaterials for the controlled release of bone regeneration activity enhancing substances like Transforming Growth factor (TGF)-beta 1, Insulin-like growth factor (IGF)-1 and bone morphogenetic protein-2 [19-22] Furthermore, hydrogels can also be used as scaffolds and carriers for osteoprogenitor [23] and other cells like chondrocytes, fibroblasts and mesenchymal stromal cells.

### Ceramics

Ceramics are a large family of inorganic/non-metallic compositions with a wide range of characteristics depending on the processing method used. They can be dense, porous or non-porous and resorbable like tricalcium-phosphate [24], porous, inert and lead to bone ingrowth like hydroxyapatite-coated porous metals, or dense, non-porous, surface active materials, that attach to the bone by chemical bonding like hydroxyapatite. In this chapter only the most frequently used ceramics are cited as examples.

Calcium phosphates represent a group of materials, where their properties depend on the calcium-phosphate ratio and modification of crystallinity and porosity. They are biocompatible, osseoconductive and degradable [25, 26] by extracellular dissolution of the calcium orthophosphate material [27] and by a cell mediated resorption [28] similar to the bone remodelling or bone substitution observed with autografts [3]. Their degradation time may last up to years, depending on the type of material.

Calcium phosphate ceramic blocks are brittle, highly susceptible to fatigue fractures and therefore of limited use in complex weight bearing locations [29]. Furthermore, their preset structure may render it difficult to adapt them to local defect sites. If used in blocks, their shape may not be corresponding to the original bone defect and if used as granules it may be impossible to keep them at the implantation site.

Calcium phosphate cements can overcome this problems partially as they can be administered in paste form and injected into bone defects, which makes adaptation to local requirements very easy. In addition, they can be administered through the tissues without the need of open approaches to the injection sites. They harden without elevation of temperature. Depending on the individual composition and pH the cement setting results in brushite-cement (pH ≤ 4,2) or hydroxyapatite (pH > 4,2) [30]. There are more than 100 different formulations of calcium orthophosphate cements available, which can be divided into four classes: dicalciumphosphate dihydrate, calcium and magnesium phosphates, octocalciumphosphate and non-stoichiometric apatite cements [31].

The use of ß-tricalcium phosphate (ß-TCP) is limited by its unpredictable rate of resorption and also its biocompatibility is discussed controversary. Levin [32] reported the presence of giant cells when tricalcium phosphate was implanted, whereas Jarcho [33] to the contrary stated, that there was no foreign body cell response. On the other hand, there is consense about ist osteoconductive properties [28, 33, 34].

Dicalciumphosphate is one of the most soluble of calciumphosphate phases and can be used when quick degradation is required [35]. All cements of this group are the product of an acid-base reaction and it shows good osteoconductive properties. Despite the setting pH being quite low, tissue necrosis as a response has not been detected [31].

Hydroxyapatite is used as implant coating [36], granules [37, 38] and in block structure [39]. It has a similar chemical composition as the mineral fraction of bone and attaches close to hard tissues. It is able to fill gaps between bone and implant up to 2 mm and stimulates bone ingrowth even in osteoporotic bone [40]. In contrast to ß-TCP, hydroxyapatite bone substitutes are considered non-resorbable. However, this is only partially true since also hydroxyapatite substitutes degrade *in vivo*, albeit much slower compared to ß-TCP or brushite compositions [30, 41, 42].

Hydroxyapatite-coated implants integrate well with the bone healing process [39, 43]. This characteristic, called “osteophilic” [36], provides a good substrate for osteoblasts. The use of hydroxyapatite as a coating of implants, therefore, is quite common. However, some authors found out, that it can lead to osteolysis when it is exposed to bone marrow and soft tissues [44]. There, the hydroxyapatite wear debris is thought as the main cause for implant failure [45] as its phagocytosis stimulates the release of cytokines. Subsequently these products are held responsible for (granulomatous) inflammation, disturbance in bone remodelling and local osteolysis.

#### Natural materials:

Natural polymers such as silk, cellulose, collagen, proteoglycans, glycosaminoglycans and elastin are often quite similar to natural occurring substances which makes it easy for the body to recognize and degrade these materials by physiological mechanisms [46]. On the other hand they are immunogenic and the technological manipulation to avoid tissue reactions are elaborate and sometimes cost intensive.

## HOST RESPONSE

Implanted biomaterials are always recognized as foreign by the body, independent on how elaborate the biocompatibility was previously tested. All medical devices and prostheses implanted in connective tissue immediately induce an initial host response to act against the foreign body. The type of implant-tissue response can be graded according to Hench [24]: if the material is i) *toxic*, the surrounding tissue dies, ii) *nontoxic and biologically inactive* (nearly inert), a fibrous tissue of variable thickness forms, iii) *non-toxic and biologically active* (bioactive), an interfacial bond forms, and iv) *non-toxic and dissolves*, the surrounding tissue replaces it.

This acute inflammatory reaction resembles in large the normal wound and fracture healing process [47] and consists of cellular and molecular components. The magnitude [48] and duration of the inflammatory process has a determining influence on the stability and compatibility of the implanted medical devices.

The foreign body reaction starts within seconds or minutes after tissue contact [49] with a conditioning film of glycoproteinaceous materials called “opsonins” on the surface of the implanted device [50]. Albumin, fibrinogen, immunglobulin G and complement components are the most abundant proteins on the surface of polymers [51]. While the deposition of fibrinogen and mainly immunogloblins is considered as an active process and tissue response to the foreign material, the deposition of albumin probably can be ruled out as being part of this reaction, because it seems to more passivate the surface of implants [49]. Nevertheless, some authors found out, that the entire protein layer seems not to be critical for the immune response as either IgG deficient or complement depleted mice showed a regular reaction against foreign bodies [52].

This layer, recognized by the integrin receptors present on neutrophils and macrophages, plays an important role since it converts the implant into a biologically recognizable material [53]. It initializes monocyte attraction and migration through the endothelium through mast cell activation and associated histamin release as the next step of host response [54].

This is followed by a fibroblast invasion and synthesis of extracellular matrix through these activated fibroblasts [55]. It ends up in an inner layer of macrophages and/or foreign body cells with an outside secondary zone of layered fibroblasts and connective tissue [56] surrounding the implanted material. This reaction is unique and does not seem to depend on the type of implant [57]. The magnitude of the periprosthetic or peri-material reaction and the thickness of the inflammatory layer is said to be an index for the biocompatibility of the implant [58] as it depends on the chemical and topographical nature of the surface of the device.

Most of the routinely applied biomaterials have excellent characteristics related to biocompatibility in bulk form. Sometimes the foreign body reaction is clinically first seen when the implant is disintegrated [59-61]. The failure of implants seems to be connected with the interfacial accumulation of wear particles in case of metals and degradation products in combination with biodegradable materials [27, 62-64]. The discussion about biocompatibility is controversial [58, 65, 66], but there is a certain agreement, that the *extent *and *intensity* of tissue reaction defines the biofunctionality of an implant, rather than the response by itself [67]. In other words: not the lack of host reaction, but the appropriateness of the answer is important.

The question addressed in this article was, how this appropriateness could be defined through the appearance of different cells in the implantation site.

## CELLULAR REACTION AFTER BIOMATERIAL IMPLANTATION

**Fig. (1):**
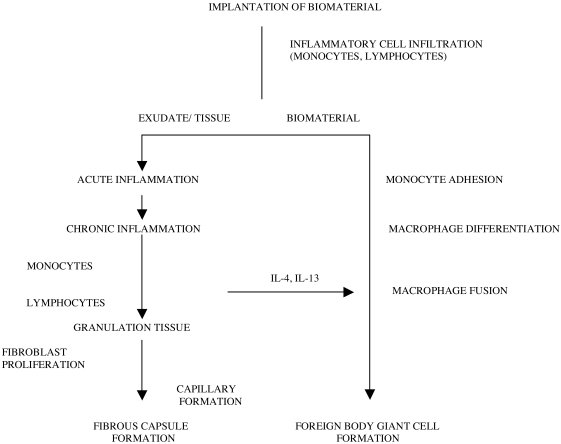
Inflammatory rection and foreign body giant cell reaction (Modified after [69]).

In bone healing processes connected with the implantation of foreign materials a specialized group of cell types with different characteristic abilities can be found (Fig. **1**).

The first cells attaching to the implanted material are the *fibroblasts*. They produce immature collagen that is laid down onto the surface of the implant [51, 55, 56, 68, 69]. These cells are recruited from the mesenchymal tissue surrounding the implanted material or fracture site upon release of signal transduction molecules of the resident bone and hematoma cells. These signals are also responsible for initiating the cascade of bone formation and resorption pertinent to fracture and/or defect healing, resp. bone remodeling [69].

**Fig. (2a,b):**
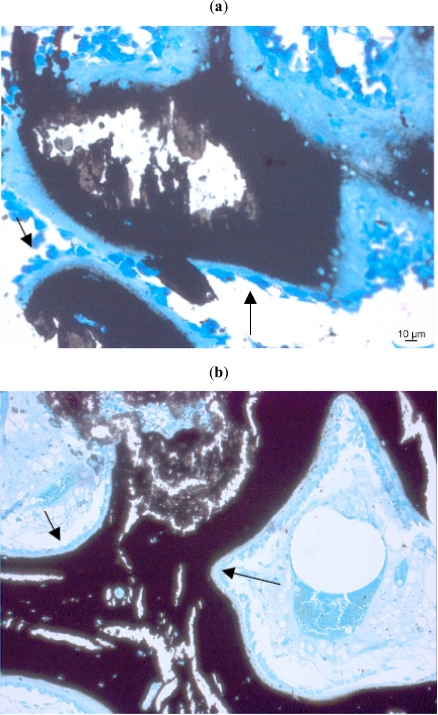
Seams of osteoblasts (arrows) close to a natural bone substitute (Kossa 20x, 10x).

*Among the bone forming cell types, osteoprogenitor cells* are detected at the inner layer of the periosteum. These cells can differentiate into osteoblasts under the influence of Bone morphogenetic protein (BMP)-2, which apart from osteogenesis also stimulates angiogenesis in bone healing. Under the condition of low oxygen tension the progenitor cells may also differentiate into chondrogenic cells. *Osteoblasts*, derived from osteoprogenitor cells synthesize the organic components of the bone, such as collagen, proteoglycan and glycoproteins (Fig. **2**). After their differentiation they express bone specific alkaline phosphatase (ALP), which therefore, is a late marker of bone formation. *Osteocytes* derived from osteoblasts are mature bone cells, that became trapped in their lacunae. Osteocytes keep in contact to each other by cytoplasmatic processes, through which ions and small molecules can move between the cells [70].

**Fig. (3a,b):**
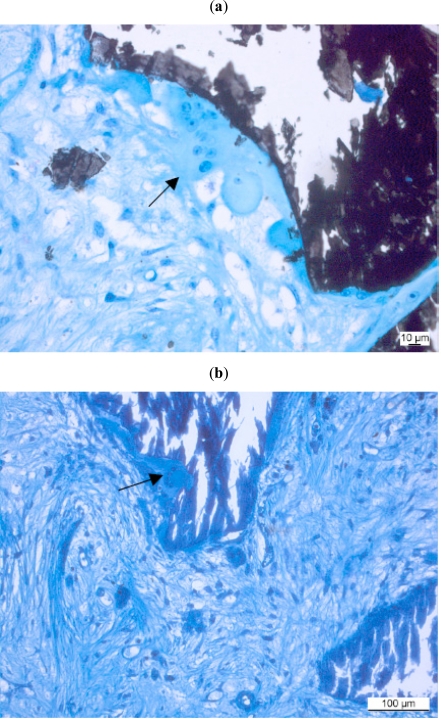
Osteoclasts degrading a natural bone substitute material (arrows). (Kossa 20x, Toluidinblue 10x).

Cells responsible for bone (or material) resorption are connected to the osteoclast lineage. The precursor cells of *osteoclasts* come from the bone marrow and are called granulocyte-macrophage progenitor cells [71]. They are thought to derive from the blood macrophages. Upon stimulation by local signalling molecules, mediators and cytokines (such as receptor activator of nuclear factor-B ligand (RANKL), prostaglandin E2, interleukin (IL)-1 and 6) these originally mononuclear cells fuse to become multinucleated cells generally found on mineralized surfaces in bone (Fig. **3**) Osteoclasts are responsible for resorbing bone, especially the woven bone which first appears after a bone wound has been produced, such that this immature substitute can be replaced by lamellar bone. It is not always possible to distinguish osteoclasts from foreign body cells (FBGC) with certainty. Osteoclasts resemble foreign body giant cells morphologically but have calcitonin receptors on their surface. To distinguish osteoclasts from FBGCs immunostaining of osteocalcin receptors would, therefore, be the modern method of choice. Formerly, special stainings for the tartrate-resistant acid phosphatase (TRAP): TRAP [72] were routinely performed for this task. Some authors found out, that the TRAP-coloration was not entirely specific for osteoclasts [73]. However, technical problems with immunostainings in bone especially in larger animal species and in combination with biomaterials, where bone samples have to be embedded in plastic sections, make it impossible to successfully use osteocalcin antibodies for osteoclast identification.

Osteoclasts can carry out the highly specialized function of lacunar bone resorption, but it is generally believed that they do not phagocytose particles of biomaterials at the bone-implant interface [74]. However other investigations show, that they are capable of phagocytosing both polymeric and metallic biomaterial particles. This lead to the conclusion, that not all multinucleated cells, that contain wear particles and can be found next to osteolysis are FBGC [75].

In the line of defense, lymphocytes and *plasma cells* produce antibodies to protect the body against foreign antigens, they are seen in greater numbers in areas of more chronic inflammation and where foreign substances have entered the tissue [56, 65, 68, 69, 76-80] (Fig. **1**). *Neutrophils* participate in the foreign body reaction by releasing lytic enzymes [81]. Usually neutrophil polymorphs are found in the immediate period after lesions were created for wound debridement, but mark the presence of (sub-)clinical bacterial infection if found later in the wound healing period.

In addition, two other cell types are also especially interesting considering cellular defense mechanism to foreign material: macrophages and foreign body giant cells (FBGC). There is no general agreement in the literature about their character of being inflammatory and thus, negative for the process, or just belonging to a normal response in degrading materials.

*Macrophages* derive from the mononuclear phagocyte system. All members of that system arise from a common stem cell in the bone marrow, possess lysosomes and are capable of phagozytosis. In the first few days after a fracture has occurred or a bone defect has been produced, granulation tissue grows in between the fracture ends or edges respectively. As mentioned above, neutrophil polymorphs are the type of cells which can be found most frequently in this period. The macrophage derived interleukin-1 may cause neutrophil infiltration, induce angiogenesis and antibody production and lymphokine synthesis [82]. Later more chronic inflammatory cells like macrophages are found, which remove red cells, necrotic fat and tissue debris [76]. These are transformed monocytes which arrive at the implantation site *via *a complex pathway of chemotactic and chemokinetic agents like mast cells/histamine [54], and Tumor Necrosis Factor (TNF) -alpha [83] release, complement factors, lymphokines, chemokines, platelet factors, leukotrienes and eventually bacterial fragments [84]. They adhere to the biomaterials *via *several adhesion ligand-receptor superfamilies [80], such as with an integrin binding [68]. Certain biomaterial-adsorbed proteins promote monocyte adhesion. The most active in this regard are fibrinogen, fibronectin and immunglobulin G [85-87].

After adhesion they transform into macrophages which are characterized by cell enlargement and an increased secretion of inflammatory mediators (cytokines and chemokines), an increased expression of membrane proteins (e.g. integrins) [88, 89] and the expression of angiogenic and other growth factors [80]. The released factors attract different cell types like additional macrophages, neutrophils, fibroblasts and other cells [89-92].

Macrophages are cells that secrete factors to promote physiological wound healing. Some of the macrophages may also function as accessory antigen-presenting cells [93]. On the other hand, they can be the central cellular mediators of the chronic inflammatory response to foreign materials [56] by secreting monocyte chemoattractant protein-1 (MCP-1) contributing to the development of the foreign body reaction [94], disturbing wound healing and ultimately contributing to implant failure.

**Fig. (4):**
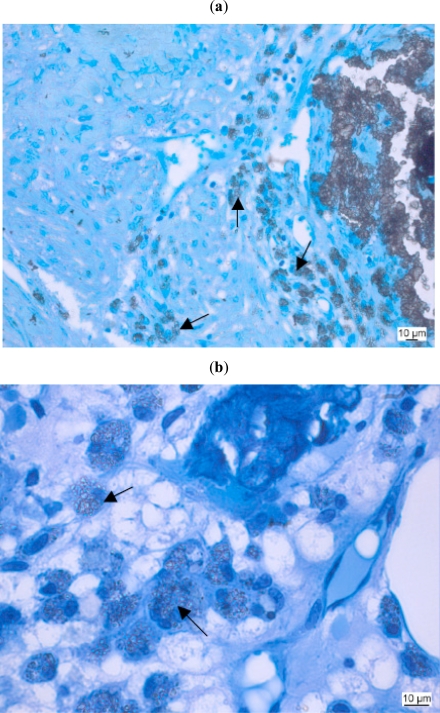
Biocompatibility study of a biomaterial as bone substitute in sheep bone. Macrophages (arrows) with ingested foreign material. (**a**: Kossa 20x, **b**: Kossa 40x).

Macrophages phagocytose damaged cells, cellular debris and foreign substances and digest the ingested material by hydrolytic enzymes in their lysosomes. While this mechanism functions for several biomaterials in the same way, their enzymatic apparatus is not able to degrade synthetic polymers [95]. Some authors consider the presence of macrophages around or near biomaterials to be part of a chronic inflammatory reaction, whereas others relate to them as part of the normal degradation behaviour, at least in case of degradable materials in bone [27, 62, 96, 97] (Fig. **4**).

Macrophages also modulate the tissue reaction through production of interleukins, growth factors and other bioactive agents and most importantly they are the precursors of osteoclasts and foreign body giant cells. The role of RANKL and what triggers the fusion and further differentiation into osteoclasts or foreign body giant cells is not entirely clear [96, 97].

Mechanical wear of implants (the smaller the more) [45] activate macrophages to phagocytosis which in turn induces secretion of TNF-α, IL-1ß, IL-6 and prostaglandin (PGE)_2_ [45, 98] that stimulates differentiation of osteoclast precursors into mature osteoclasts [99]. This effect leads to bone resorption and in excess can induce implant failure [100]. While in debate whether osteoclasts contain foreign material in their cytoplasm, it also has been shown, that macrophages, which have phagocytosed particles, are capable of osteoclast differentiation [101].

Interaction of macrophages and lymphocytes are complementary. It is likely that the adhesion of macrophages to a surface also is the initial signal to activate the lymphocytes which in turn release molecules that furthermore influence macrophage activity [77] and fusion [81, 102-104]. Therefore, the presence and activity of lymphocytes may be a determining factor in excessive resorption behaviour or ultimate biocompatibility questions.

Macrophages also modulate in the process of tissue repair. Since they derive from the vascular system, a good vascularity, therefore, is one of the important factors [105]. Animals depleted of macrophages or having received antimacrophage monoclonal antibodies show deficient wound healing [106].

*Foreign body giant cells* are present in cases of bone defect healing with or without autologous bone grafts and play a significant role if biomaterials are applied. In case of biomaterials and bone controversy exists in whether they are part of normal bone healing or material degradation, or whether they play a significant role in issues of bioincompatibility.

Under chronic inflammatory conditions and if the matter to be disposed is very large (bigger than the diameter of a macrophage [27], than 80 µm [95], 5µm [44], 12 µm [35]), or indigestible for osteoclasts [107, 108], several macrophages fuse to form a foreign body giant cell [109]. Anderson [69], in contrary, has the opinion that „the presence of mononuclear cells, including lymphocytes and plasma cells, is considered chronic inflammation, whereas the foreign body reaction with the development of granulation tissue is considered the normal wound healing response to implanted biomaterials“. His view in regard to foreign bodies is similar to Lassus *et al*. who considers macrophages to be part of the normal degradation process [96, 97].

The mechanism of cellular fusion of macrophages to FBGC is similar to phagocytosis and is mediated by several mediators [110, 111], but little is known regarding the biological responses which are considered to influence the transition to FBGC development [69].

It can lead to very large cells (up to 1 mm^2^) with hundreds of nuclei [112, 113]. FBGCs are also generally observed in granulomas induced by bacterial pathogens, such as in tuberculosis or trichinellosis, which is probably the main reason for the negative association with their presence in tissue.

Cytokines like interleukin-4 and 13 are known as potent inductors of macrophage fusion into FBGC [103, 114, 115]. They also play a central role in cellular reactions which cause bone lysis around implants [116] as they modulate the balance between osteoblasts and osteoclasts [117]. The injection of anti-interleukin-4-antibody significantly decreased FBGC density on polyetherurethane ureas *in vivo* [102].

Foreign body giant cells act to concentrate phagocytic and degradative activities at the host-implant interface. They ingest and dissolute implanted material intracellularly or by the release of degradative agents like lysosomal enzymes and reactive oxygen intermediates (ROIs) at the ventral cell surface [118] in response to certain stimuli, whereby their phagocytic capacity can be as effective as macrophages [119]. They also can directly contribute to osteolysis by differentiation into TRAP-positive osteoclast-like cells [120-122].

**Fig. (5):**
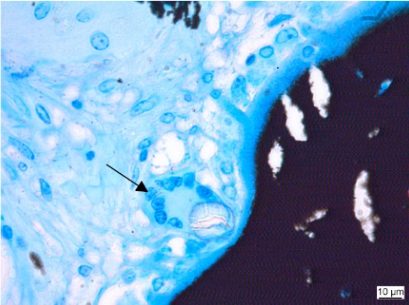
Foreign body giant cell (arrow) close to implanted material. (Kossa 40x).

FBGC rapidly differentiate after the implantation and progressively decrease with time [123]. Their size depends on the intensity of the inflammatory response [124]. They resemble osteoclasts morphologically, such that both are multinucleated, are found near implant/bone contact and have a cytoplasm with many vacuoles (Fig. **5**). FBGCs contain great numbers of mitochondria of various size and oval or round nuclei. Rough endoplasmatic reticulum is found throughout the cytoplasm [125]. The osteoclast on the other hand is defined as possessing a resorbing apparatus consisting of ruffled border and clear zone, expression of the tartrat–resistant acid phosphatase and the expression of calcitonin-receptors [125]. However, it has to be kept in mind that other authors question whether the TRAP-epitopes as well as other markers (such as calcitonin receptors) are specific for osteoclasts [73].

One of the most important aspects in the evaluation of biomaterials is the degradation resistance, with the exception of the class of biodegradable polymers, that rely on enzymes, acid or ROIs for degradation of the polymer matrix [126]. The discussion about the role of FBGCs is controversary with the main question being whether the presence of FBGCs near an implant is just a sign of biodegradability [126], therefore a part of normal bone healing and resorption after the implantation of a bone substitute, or a sign of insufficient biocompatibility, inflammation and implant failure? Both sides have valuable arguments ready which are outlined below.

The arguments in favour of the FBGC are that i) these cells are part of the normal wound healing response to implanted inert or biodegradable biomaterials [56, 127], ii) their presence does not impair bone formation [31], iii) the presence of FBGCs indicates a *low degradability* of the implanted substance [125, 128, 129], iv) FBGC in the absence of other inflammatory cells show a *good biocompatibility* [107], and most importantly v) macrophages and FBGCs mediate material resorption and fragmentation of biodegradable implants [125, 129-131]. The latter is supported through the fact that resorbed material could be seen in intracytoplasmatic vacuoles by transmission electron microscopy [132]. Furthermore, the implantation bed of resorbable poly-L-lactide (PLLA) plates and screws showed a “foreign body reaction without signs of inflammation”: only a few polymorph nuclear leucocytes were present and the remnants of the plates and screws were surrounded by connective tissue with macrophages, foreign body giant cells and fibrocytes. The foreign body reaction was thought to be evoked by very small particles (22 µm) of disintegrated PLLA plates and screws [61] and the amount of the degraded material seems to influence the intensity of the foreign body reaction [133]. This view, that FBGC are responsible for the degradation of biomaterials, is further supported by the analyses of retrieved implants showing material surface cracks directly under adherent FBGC [113]. If macrophages and foreign body cells are part of the normal wound healing process, the foreign body cells may persist for the lifetime of the implant, it is not known if activated or quiescent [69]. Biocompatibility may be in jeopardy, if the presence of a large mass of disintegrated material seems to “exceed the local tissue tolerance” [61]. Here, the question arises where the line has to be drawn for local tissue tolerance?

The arguments pointing towards FBGC being a a bad sign for tissue tolerance are that i) the fusion of specialized macrophages is induced by *poorly tolerated* foreign bodies [78, 112, 134], ii) avoiding monocyte or macrophage adhesion and FBGC formation, inflammatory degradation could be minimized [135], iii) the presence of macrophages and FBGC is associated with structural and functional *failure *of the implant [136], and iv) FBGC seem to concentrate the phagocytic and degradative activities at the tissue-material interface and therefore are *responsible for the damage and failure of the implant*. For the latter the authors tested coating the surface of an implant with a material that promotes programmed cell death to inhibit the adhesion and fusion of macrophages into FBGCs [137].

*Fibrous capsule formation* close or around an implanted biomaterial is frequently seen. The reasoning behind it is that if the implanted biomaterial cannot be ingested by macrophages and FBGCs (“frustrated phagocytosis”) the next best protection for the host seems to be the isolation of the foreign object. This can be achieved best by a layer of FBGC in a fibrous, quite avascular capsule limiting further interaction between host and implanted device. The capsule type depends on the secreted cytokines [95, 138], the extent of injury or defect created and the amount of provisional matrix [69].

On one hand this fibrous capsule may indeed downsize the inflammatory reaction, but on the other hand in osseointegration processes can lead to device failure and restricted nutrient supply. Poor tissue device contact can lead to infection. A fibrous layer (so called interface membrane) between methylmethacrylate and an implant has shown to be morphologically synovial-like [55]. In well fixated or coated [139] implants the interfacial membrane is thin with only some macrophagic aggregates. The quantity and quality of the fibrous scar tissue depends on the implantation technique and the prosthetic material used [11-13, 55].

## INFLUENCES ON BIOCOMPATIBILITY AND FOREIGN BODY RESPONSE

The type of biocompatibility and foreign body response depends on the chemical composition/surface character, morphology, localization of the implant the surgical technique and mechanical loading [140, 141]. Besides that, degradability, hormonal and humoral influences play an important role.

### Implant

#### Surface morphology

The implant surface-tissue interface is the most important relationship for biocompatibility *in vivo* especially in metallic implants but also in other biomaterials [81] as the surface character influences the nature and magnitude of the foreign body reaction [142]. The biocompatibility of an implant and the irritation of the surrounding tissue depends on different parameters like hydrophobicity/hydrophilicity, wettability, surface charge, polarity, surface energetics, mobility of the surface molecules and smoothness. Thereby it seems to depend more on physical attributes than on the implant`s chemical composition [65].

The hydrophilic surface of hydrogels for example has a small amount of interfacial free energy to react with body fluids and this results in a low tendency for proteins and cells to adhere and grow onto these surfaces [143, 144].

In the contrary Eriksson *et al*. [145] compared the healing response between hydrophilic and hydrophobic implant surfaces and found out, that the difference in the adherence of viable cells can be seen only initially. A certain roughness of an implant prevents excessive tissue motion and therefore results in a relatively thin soft tissue layer in comparison to the polished surface of the common stainless steel implants [146]. On the other hand the resulting surface enlargement can increase the risk for corrosion.

Cells do not adhere directly to the surface of synthetic implanted materials, but to extracellular matrix proteins. Implants coated with phosphorylcholine have been shown to reduce this protein adsorption and this resulted in a lower inflammatory response and a lower fibrous capsule thickness [139].

Some authors refer to implant failure as a „small particle disease“ especially with metallic devices, because the interfacial membrane is full of implant debris [63]. The physical characteristics of those particles play an important role in the resulting inflammatory reaction [45]. To avoid excessive particle formation mechanical biostability is especially important in implants that are planned to remain in the body for a long time [11-13].

Materials composed of elements near to calcium and carbon in the periodic system are more biocompatible over time than others, because the body is composed mostly of those elements and water [81, 147]. If implants release drugs, the original situation may change again and the biocompatibility of the devices is affected by the bioactive compounds they deliver to the surrounding tissue [148].

Hochuli-Vieira *et al*. [127] compared the body’s reaction on titanium plate and PLLA/polyglycolic-acid (PGA) implant fixation after mandibular osteotomy in rabbits. The PLLA/PGA plates and screws were partially degraded after 30-60 days. The implants were wrapped in connective tissue with some macrophages and FBGCs. No specific inflammatory reaction was seen, but “some macrophages were in contact with the screws” or “some scarce giant cells and macrophage cells around them” after 30 days of implantation.

An essential prerequisite for osteoconduction is the direct, stable and extensive enough contact between host bone and the implanted material. One way to optimize the biocompatibility of an implant could be to coat the surface with a biocompatible film [149]. However, the best biocompatibility may be achieved when tissue can grow into the pores of the implanted material. As pores smaller than 50 µm exclude macrophages to clear bacteria away, pore size, therefore, should be about 60 µm to avoid bacterial infection [30, 62].

On the other hand high-surface-to-volume-implants as porous devices are said to have higher ratios of macrophages and FBGCs in the implant site [69]. Bone ingrowth seems to increase with the size of the pores [150], at least initially but not in the long run. Pore dimensions of at least 10 µm seems to be necessary for connective tissue ingrowth [151]. A minimal pore size of more than 100 µm [24], even of 200-400 µm has been recommended [152], the latter being close to the average size (223 µm) of the human osteon.

#### Degradability

Most synthetic polymers are degraded through hydrolysis, whereas most biopolymers such as collagen are degraded through enzymatic attack. An optimal degradable scaffold material should be degraded and resorbed at the same rate as the tissue grows into the implant and replaces it with natural bone [153] as nondegraded polymers are preventing complete ossification. Polylactides, for example, degrade slowly through hydrolyzation, and some residues can be found in the region of implantation up to 2 years [128].

Bone healing studies have shown, that there is a “bone signal window” about 7 to 12 weeks after injury. After that time the natural bone healing signal disappears and a fibrous tissue scar will be the result. Therefore, the authors conclude that an implant should degrade up to 12 weeks, at least in bone [154].

After the implantation of Ca/P-apatite-coated polylactone sponges and non-coated sponges as a control FBGC were observed in close contact to all implants. Their number seems to correlate with the amount of non-degraded and not on the composition of the material [128]. As mentioned above, it may be difficult to draw the line which rate of degradability and subsequent FBGC formation is still tolerable as part of normal wound healing processes and where bio*in*compatibility issues start.

Any material implanted elicits a defensive process, but modern biomaterials can be well accepted by the tissue in which they are implanted. Their particular breakdown products on the other hand can induce a severe inflammatory reaction [63]. The cellular activity while PLLA plates are resorbed showed a dense layer of macrophages in the first few weeks and in the end stage of the resorption process after 104 to 143 weeks [155]. The changes in biomechanical properties and morphology due to the degradation may intensify the foreign body reaction [156]. It has been stated by several authors that the accumulation of macrophages is a general biomaterial phenomenon related to degradable biomaterials [157-160].

#### Localisation

Biocompatibility depends on the surface structure of the medical device and, of course, also on the tissue, in which it is implanted [64, 161]. Medical devices implanted in different tissue types provoke a different reaction. Here, the statement of Williams should be remembered, that “the unique cicumstances pertaining to individual cases have to be considered when defining biocompatibility” [162].

### Surgical Technique/Implantation Model

There are two different models representing different principles to test novel materials in bone: **a wound model**, where spontaneous healing will occur in a short time, provides information about osteocompatibility and inflammatory potential of a given material; and a model of large **osseous defects** [163], that do not heal spontaneously.

The correct placement of an implant is of utmost importance, as post implantation mobility leads to chronic inflammation with the development of a thick fibrous capsule with all its disadvantages [11-13].

### Mechanical Loading

Bone is subjected to high strain [164, 165]. In its function of load bearing continuous microdamage to bone occurs [166] that constantly demands structure remodelling. As described earlier this remodelling is mostly coordinated by bone forming cells (osteoblast, osteocytes and periostal cells), monocyte-derived osteoclasts and multinuclear cells. Extensive micromotion between implant and host bone leads to an intermediate layer between them. One has to keep in mind that this intermediate layer, also called interface membrane, cannot be distinguished according to its primary cause such as biocompatibility and/or mechanical problems. Tissue composition and cellular components are more less identical in both cases and again, this thick and fibrous layer severely disturbs osseointegration of any implant.

### Hormones

Biomaterials inserted into bone normally elicit local and very seldom systemic responses. Therefore, the influence of systemic hormones may be negligible in most cases. Nevertheless, hormones do influence the overall reaction of the immune system of a patient or an experimental animal which consequently may even have a – albeit relatively minor-local effect on immune response. Hormon concentrations may be directly related to the intensity of the immune response and as a result also to the tissue-implant response [148, 167]. High doses of steroid hormones could have significant down regulating effects on immune response [168]. High testosteron levels and low estrogen levels seem to depress immune function [169]. Estrogens are enhancers of humoral immunity [105].

## DISCUSSION AND CONCLUSIONS

Different cell types interact and lead to specific responses. Understanding these mechanisms may allow us altering or influencing these interactions [170] and finally arrive at implant surfaces that promote greater biocompatibility. Every unnatural implant induces a natural foreign body response, thus, it is not the response by itself but its extent and intensity on which the biocompatibility of a medical device is determined. Therefore, any definition of biocompatibility related to a certain material should be based on quantitative evaluation of the cell population surrounding the implant, not just on the types of cells that occur.

The presence of macrophages and FBGCs including the development of some granulation tissue is a normal answer. For *degradabl*e materials the action of those cells are needed, as the result of an implantation otherwise would be a thick fibrous capsule around the device with all its negative consequences.

For *permanent* materials macrophages and FBGCs are responsible for the removal of wear debris that even in the best of cases cannot be prevented completely. However, a material, that remains relatively stable and unchanged in the surrounding tissue and a low amount of wear particles will not induce a permanent foreign body reaction leading to implant failure [11-13]. The body tissue seems to be capable of handling a continuous but minimal amount of wear debris without negative consequences, at least in bone. The appropriate and physiologic answer to the implantation seems to be the acute inflammatory reaction of the body with the mechanisms of macrophage and foreign body giant cell action for both permanent and degradable materials which ends up in either the bodies acceptance of the non-degradable device or the removal of the degradable foreign body as fast as it can be replaced by new bone. Only persistent inflammatory stimuli lead to chronic inflammation [69] including mononuclear cells like lymphocytes and plasma cells. Those are induced by the chemical and physical properties of the object or motion and if present in high numbers at the implant site lead to implant failure and/or rejection. In fact, the natural reaction of the body to foreign (bio) materials is similar to granuloma formation seen with infectious diseases (e.g. tuberculosis, parasites, such as trichinella, etc.,). There, granulomas are graded according to their biological activity [171]. Granulomas with low activity just there in the tissue for years without causing harm [172, 173].

However, since biomaterials are not infectious, but are either inert or fast or slowly degrading, resp. resorbing, it may be the extend of “granuloma” formation that determines their ultimate biocompatibility. A thin fibrous capsule may not be avoided and seemingly can be easily tolerated also in the bone. The same ist true for the presence of macrophages and/or FBGC. The material type, the speed of degradation and/or resorption may be responsible whether mainly macrophages alone will transport the material or whether fusion to FBGC resp. osteoclasts occurs [27, 55, 95-97, 117, 138]. Even low numbers of lymphocytes and plasmacytes occasionally present within the fibrous tissue surrounding the implant may not seriously jeopardize material acceptance, whereas the combination of a thick fibrous capsule, high numbers of macrophages, osteoclasts and/or FBGC and the presence of lymphocytes and plasmacytes can be considered a tissue reaction associated with bioincompatibility.

Even though a low tissue reaction to biomaterials may be tolerable, improvement of biomaterial surfaces should still be attempted. Future work should be directed towards *i)* the inhibition of the (immune) cell adhesion to (non-degradable) implants by adding antibodies that bind to the integrin receptor that recognizes the adhesion protein ii) the investigation of surface-chemistry for implanted (degradble or non-degradable) biomaterials, that modulate short- and long-term adhesion of monocytes and macrophages and FBGC fusion to the special needs of each type of implant, iii) the option to manipulate macrophage behaviour by harnessing some of the beneficial functions, like the expression of angiogenic and other growth [53].

Tissue responses may be further ameliorated and be kept to a minimum. Nevertheless, macrophages and FBGC may never be eliminated and may always be part of tissue response towards degradable materials.
